# Auditory Evoked Potentials Across Cognitive States: Awake, Sleep, Sedation, and Anesthesia

**DOI:** 10.3390/medsci14060618

**Published:** 2026-09-30

**Authors:** Zhibin Zhou, Nayana Singh, Miguel Padilla, Kevin Goehl, Hong Liu, Nathaen Weitzel

**Affiliations:** 1 Department of Anesthesiology and Pain Medicine, School of Medicine, University of California, Davis, Sacramento, CA 95817, USA; hualiu@health.ucdavis.edu (H.L.); nsweitzel@health.ucdavis.edu (N.W.); 2 Reno School of Medicine, University of Nevada, Reno, NV 89557, USA; nayanas@nevada.unr.edu; 3 Department of Neurobiology, Physiology and Behavior, College of Biological Sciences, University of California, Davis, CA 95616, USA; migpadilla@ucdavis.edu; 4 KBO Systems, Aurora, CO 80016, USA; kevin.goehl@kbosystems.com

**Keywords:** auditory evoked potential, auditory brainstem response, mismatch negativity, P300, steady-state auditory evoked potential, sleep, sedation, anesthesia, consciousness

## Abstract

Auditory evoked potentials (AEPs) comprise a family of electrophysiological responses that follow acoustic stimulation from the auditory nerve and brainstem to thalamocortical and association networks. Their interpretive value lies in the combination of millisecond temporal resolution and hierarchical sensitivity: early responses mainly index the fidelity and timing of ascending sensory conduction, whereas later responses increasingly depend on cortical context, attention, prediction, and behavioral relevance. This review develops a state-aware framework for interpreting AEPs during wakefulness, natural sleep, pharmacologic sedation, general anesthesia, and impaired consciousness. We first describe the ascending auditory pathway and relate approximate latency ranges to distributed neural generators. We then examine how attention and task engagement modify early cortical processing; how non-rapid eye movement and rapid eye movement sleep preserve some forms of acoustic analysis while reorganizing or suppressing others; and how anesthetic effects vary with response latency, drug concentration, and cortical hierarchy. The review also distinguishes transient event-related potentials from steady-state auditory evoked potentials (SSAEPs), and explains why phase-locked activity can be recovered by trial averaging whereas non-phase-locked induced activity requires single-trial time-frequency analysis. Separate sections address cross-species translation; click, chirp, oddball, steady-state, and naturalistic speech paradigms; as well as clinical applications in hearing assessment, intraoperative monitoring, anesthesia, and disorders of consciousness. The central conclusion is that an AEP is interpretable only when its generator, stimulus, analysis domain, recording conditions, and cognitive state are considered together.

## 1. Introduction and Scope

Auditory evoked potentials are voltage fluctuations recorded at the scalp or directly from neural tissue after an acoustic event. The first reproducible far-field human brainstem responses demonstrated that very small, time-locked signals generated deep in the auditory pathway could be recovered noninvasively by repeated stimulation and averaging [1,2]. Soon afterward, systematic studies showed that the auditory response is not a single waveform but a succession of components extending from the first milliseconds after a click to late positive potentials several hundred milliseconds later [3]. This latency hierarchy remains useful, but latency alone is not a complete taxonomy: components also differ in neural generator, dependence on stimulus regularity, susceptibility to attention, and vulnerability to changes in arousal.

The distinction between predominantly stimulus-driven (exogenous) and context-dependent (endogenous) responses provides a second organizing axis [3,4]. Early waves are strongly constrained by stimulus timing and peripheral conduction, although longer-term experience and descending control can also influence subcortical encoding. Later cortical components depend increasingly on how the current event relates to preceding events. Selective attention increases cortical responses to attended sounds [5,6], rare or uncertain targets evoke the P300 family [7], and violations of an auditory regularity can produce mismatch negativity (MMN) even when the auditory stream is not the focus of voluntary attention [8]. These findings suggest that AEPs provide a bridge between clinical neurophysiology and cognitive neuroscience.

Cognitive state is therefore not a nuisance variable to be averaged away. Wakefulness, drowsiness, non-rapid eye movement (NREM) sleep, rapid eye movement (REM) sleep, sedation, and general anesthesia alter neuromodulatory tone, background oscillations, behavioral responsiveness, and effective communication between cortical regions. The same physical sound can consequently produce an intact auditory brainstem response (ABR), an attenuated middle-latency response (MLR), a residual deviance response, or no task-dependent P300. A preserved early component shows that sound activated the auditory nerve or brainstem level being tested; it does not establish intact conscious perception. Conversely, a late context-sensitive response can reveal processing not evident from behavior, especially in a patient who cannot communicate. Throughout this review, state denotes a specified arousal, behavioral, or pharmacologic condition—wakefulness, stage-scored sleep, drug-defined sedation or anesthesia, or impaired consciousness—rather than an undifferentiated label.

This review uses that hierarchical dissociation as its central framework. It asks four practical questions: (1) which generator populations contribute to each response; (2) which components are relatively preserved or strongly modulated across cognitive states; (3) which findings from animal AEP studies can be reasonably translated to humans; and (4) which stimulus and analysis choices are required to test a particular level of processing. The scope includes ABR, MLR, N1/P2, MMN, P300, frequency-following responses (FFRs), and SSAEPs. Otoacoustic emissions are faint sounds generated mainly by cochlear outer hair cells and recorded in the ear canal. They are discussed only as an independent measure of cochlear function, not as neural AEPs or as a source of AEP noise. Purely spontaneous EEG is included only when it provides a complementary measure of state. Animal studies are included when they permit causal lesions, laminar recordings, or controlled pharmacology that are not feasible in humans. They are used to test mechanisms, not to assign human conscious experience; translation requires matched stimuli, conserved computations, and explicit attention to species, anesthesia, and recording geometry.

The framework is probabilistic rather than absolute. Latency ranges overlap; scalp peaks combine activity from multiple sources; and pharmacologic effects depend on the agent, concentration, stimulus, montage, and physiologic conditions. Accordingly, terms such as “preserved,” “attenuated,” and “absent” describe tendencies observed in groups under defined acquisition conditions. They should not be treated as universal properties or used alone to classify an individual patient’s state. This distinction is especially important when AEPs are used to infer awareness or prognosis.

Prior reviews have often concentrated on individual components or on anesthesia-specific monitoring. The present review instead integrates generator hierarchy with state physiology, separates local deviance from global-rule processing, distinguishes phase-locked evoked activity from induced activity, and connects these distinctions to a clinical translation matrix [4,9].

### Review Approach

This is a narrative review. PubMed/MEDLINE was searched from database inception through 25 September 2026 using combinations of auditory evoked potential, ABR, MLAEP, MMN, P300, SSAEP, ASSR, and FFR with wakefulness, attention, sleep, sedation, anesthesia, consciousness, disorders of consciousness, intraoperative, newborn, and pediatric. Reference lists of relevant articles and reviews were also screened. English-language human and animal original studies, methodological papers, and reviews were included when they directly informed AEP generators, state modulation, acquisition or analysis, or clinical translation; reports without an auditory evoked measure or with insufficient relevance to those questions were excluded. Selection was purposive rather than exhaustive, and no formal risk-of-bias assessment or meta-analysis was performed. The final synthesis cites 63 sources. Candidate records were not logged prospectively, so a PRISMA-style screening count is not available.

## 2. Neurophysiological Foundations of AEPs

### 2.1. Ascending Auditory Pathways and Distributed Generators

**Figure 1:**
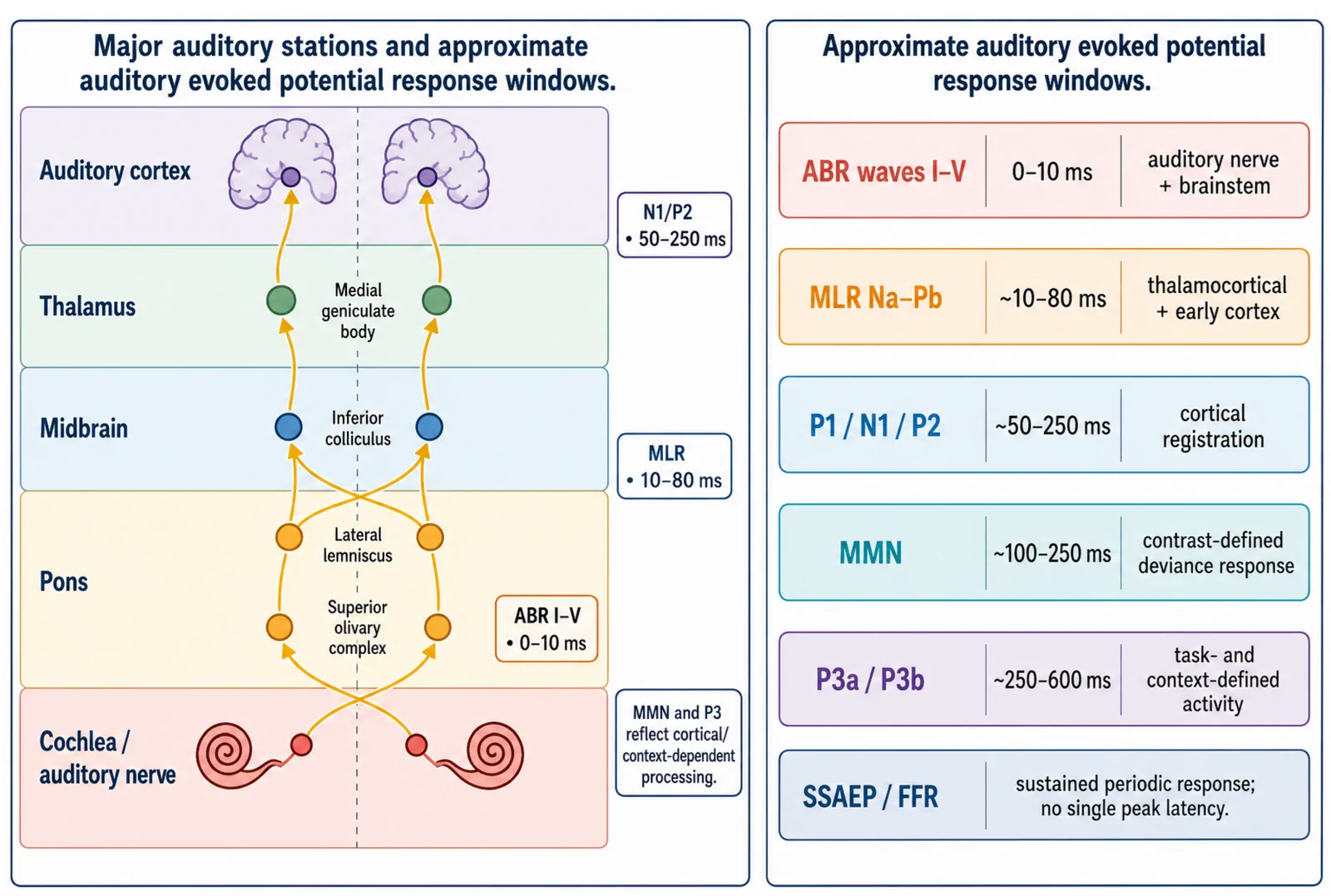
Major auditory stations and approximate AEP response windows. The anatomy is a conceptual synthesis, and the transient-response windows are not drawn to scale. Scalp components reflect distributed generators and overlapping windows; SSAEPs are sustained periodic responses rather than single-latency peaks [11,12,13,14,15,16,19].

Inner hair cells transduce basilar-membrane motion into neural activity, and auditory-nerve fibers encode the timing and frequency content of that activity. The ascending stream then passes through the cochlear nuclei, superior olivary complex, nuclei and fibers of the lateral lemniscus, inferior colliculus, medial geniculate body, and auditory cortex. Beginning in the brainstem, projections are substantially bilateral (Figure 1), so input from either ear reaches both sides of the central pathway. This organization supports neural computations for binaural integration and spatial hearing but prevents a simple one-wave-to-one-nucleus mapping. Human lesion studies nevertheless established a clinically useful relationship between ABR timing and lesion level: abnormalities in early peaks and interpeak intervals can help distinguish peripheral or lower-pontine dysfunction from more rostral conduction abnormalities [10].

Controlled experiments in cats clarified why the clinical relationship between ABR timing and lesion level is informative but not one-to-one. In a three-part series, investigators used selective kainic-acid lesions of the cochlear nucleus or superior olivary complex, related the lesion sites to far-field waveform changes, and integrated the results with anatomical and physiological evidence [11,12,13]. In this cat nomenclature, P2, P3, P4, and P5 are early brainstem auditory peaks, not the human cortical P2 or P300. The earliest neurophysiologic waveform extrema arose from cells peripheral to the cochlear nucleus; cat P2 was associated chiefly with cochlear-nuclear populations; cat P3 reflected mixed cochlear-nuclear and superior-olivary activity; and cat P4–P5 reflected distinct parallel pathways involving cochlear-nuclear and bilateral superior-olivary populations. A scalp peak can include stationary currents in aligned cell populations, activity propagating along fiber tracts, and downstream effects of interrupting an earlier relay. Generator labels should therefore be treated as dominant associations rather than exclusive sources.

Temporal synchrony is as important as anatomy. A neural population contributes strongly to a scalp-recorded far-field response when many neurons activate within a narrow time window and their current dipoles have orientations that sum rather than cancel at the scalp. In click-ABR protocols, alternating click polarity can reduce the cochlear microphonic and electrical stimulus artifact because those artifacts reverse with stimulus polarity, whereas the principal neural peaks are more stable. Click polarity can still alter neural waveform details and must be reported. Conduction delay, desynchronization, hearing threshold, stimulus presentation level, temperature, and electrode montage can change peak latency or amplitude without implying destruction of a generator.

At the cortical level, organization is more complex than a single “auditory cortex.” Human postmortem mapping distinguishes core, belt, and parabelt regions in and around Heschl’s gyrus and the superior temporal plane [14]. Direct intracranial recordings show that the earliest cortical responses usually occur in posteromedial Heschl’s gyrus, but later activity does not always proceed through a strictly serial core-to-belt-to-parabelt chain [15]. Instead, primary and non-primary fields exchange information through parallel and recurrent routes. These fields differ in response latency, spectral selectivity, and sensitivity to complex sounds, so a single scalp component can combine activity from several cortical stages.

Auditory cortical geometry also shapes the scalp signal. Much of Heschl’s gyrus lies within the lateral sulcus; synchronous postsynaptic currents in tangentially oriented pyramidal populations can therefore produce frontocentral voltage maxima rather than maxima directly over the temporal bone. The measured N1, for example, is a composite of overlapping processes rather than a single generator. Direct recordings from the human auditory cortex further place several MLR components along Heschl’s gyrus, supporting a transition from subcortical conduction to early cortical processing within approximately the first 80 ms [16].

Descending and recurrent influences complete the circuit. In humans, selective attention changes early auditory-cortical responses within tens of milliseconds [17]. In behaving ferrets, task engagement rapidly reshapes spectrotemporal receptive fields in the primary auditory cortex [18]. Corticothalamic and corticofugal pathways provide routes by which learning and expectation can also influence subcortical encoding. These examples do not imply that every early ABR peak changes with moment-to-moment attention; the magnitude and time scale of modulation depend on the response, task, and analysis. A useful working model is a predominantly feedforward early volley embedded in a recurrent system whose later components express progressively more context.

### 2.2. Components, Generators, and Time Scales

AEP components are commonly grouped by latency, but the boundaries are approximate and shift with age, stimulus presentation level, repetition rate, filtering, and nomenclature. The ABR occupies roughly the first 10 ms after stimulus onset in adults and is dominated by auditory-nerve and brainstem activity. MLR components such as Na, Pa, Nb, and Pb extend from approximately 10 to 80 ms and include thalamic, thalamocortical, and early cortical contributions. Long-latency auditory evoked potentials include P1, N1, and P2, typically between about 50 and 250 ms. Context-dependent components such as MMN, P3a, and P3b overlap these windows and are defined by experimental contrasts and cognitive function, not latency alone. Figure 1 (right panel) shows the approximate response windows, and Table 1 summarizes the terminology, generators, analyses, state sensitivity, and uses.

The click-evoked ABR reflects precise neural conduction timing. Peak amplitudes are small, so hundreds or thousands of repetitions are often required. Absolute latency is measured from acoustic stimulus arrival to a peak. An interpeak interval subtracts the latency of an earlier peak from that of a later peak in the same ear; this cancels delay shared by both peaks and emphasizes conduction between the corresponding pathway levels. Wave I is most closely associated with the distal auditory nerve, and wave V is the most robust later peak in routine human recordings. Waves II–IV may be less distinct, especially near threshold or in noisy conditions. The clinically useful inference comes from the pattern across peaks within each ear and from interaural comparisons within the same patient, not from a single textbook morphology.

MLRs bridge the brainstem and late cortical response. Intracranial recordings show that Pa and later middle-latency components have generators along the supratemporal plane [16]. Because these components depend more on thalamocortical and early cortical synchrony than the ABR, they are more sensitive to arousal and anesthetic concentration. Their measured waveform can also reflect overlap between successive neural responses at rapid presentation rates and contamination from myogenic activity, particularly postauricular muscle responses.

N1 and P2 are prominent cortical responses to sound onset, but in event-related-potential nomenclature, the term “obligatory” denotes a response elicited by stimulus onset without an explicit task; it does not mean state-invariant. The N1 family contains several overlapping processes with different scalp distributions and sensitivities to stimulus onset, repetition, attention, and arousal [4]. P2 is also shaped by stimulus probability, learning, and recording context. Changes in N1/P2 across states can therefore reflect changes in the relative contribution and overlap of several cortical processes, not the on-off state of one neuronal population.

MMN is usually estimated as the difference between responses to frequent standards and physically or relationally deviant sounds [8,20]. The subtraction tests sensitivity to violation of an auditory regularity, but unequal adaptation to standards and deviants can also contribute. An equiprobable control presents the comparison sounds with equal probability and no repetitive standard, whereas a many-standards control embeds the deviant among several varying sounds; both help separate regularity effects from adaptation. P3a is commonly associated with involuntary orienting toward novelty, whereas P3b is most robust when a participant detects or evaluates a task-relevant event. These distinctions matter in sleep, anesthesia, and disorders of consciousness because a local deviance response can persist when a later response to a global or task-defined rule is absent.

SSAEPs provide a complementary frequency-domain view. In this review, “steady-state auditory evoked potential” (SSAEP) is used consistently; the commonly used term “auditory steady-state response” (ASSR) denotes the same response class. Periodic acoustic stimulation produces neural activity at the modulation or repetition frequency. Responses may also occur at integer multiples of that frequency; these response harmonics reflect the nonsinusoidal shape of the neural waveform and should not be confused with acoustic harmonics in the carrier. SSAEP amplitude and phase are typically estimated in the frequency domain, although coherent time-domain averaging is also possible. The classic 40 Hz response was originally interpreted as constructive overlap of successive MLRs [21]. Rate-manipulation and deconvolution studies show that simple linear superposition is incomplete and that adaptation and rate-dependent circuit dynamics also contribute [22,23]. The response is sensitive to drowsiness, sleep, and anesthesia, and careful montage selection is needed to avoid postauricular muscle contamination [24]. Faster modulation rates are often used for objective audiometry because they weight subcortical generators more strongly and can be easier to detect during sleep; generator composition nevertheless varies continuously rather than switching at one frequency.

FFRs also track periodic stimulus structure, but they should not be collapsed conceptually with all SSAEPs. The human FFR can represent the stimulus fundamental frequency and individual spectral harmonics of complex sounds, including synthetic vowels [25]. Early work often interpreted the scalp FFR as brainstem-dominated, but human magnetoencephalography and intracortical recordings demonstrate cortical contributions, particularly at lower represented frequencies; the measured source balance depends on stimulus frequency and montage [26,27]. Reports should therefore specify the acoustic carrier, modulation or fundamental frequency, electrode configuration, and whether the measure is a sustained time-domain waveform or a frequency-domain estimate.

**Table 1:** AEP components by latency, generator, analysis, state sensitivity, and use.

Component	Latency and Dominant Generator	Typical Paradigm/Analysis	State Sensitivity	Principal Use
ABR [1,2,10]	0–10 ms; auditory nerve and brainstem	Click or chirp; coherent time-domain averaging	Low relative sensitivity; latency can still change	Hearing threshold, neural conduction, intraoperative monitoring
MLR [16,21,22,23,24]	10–80 ms; thalamocortical and early auditory cortex	Transient clicks/tones; peak amplitude and latency	Moderate to high	Early cortical integrity and anesthetic pharmacodynamics
N1/P2 [4]	50–250 ms; auditory cortical networks	Sound onset or change; ERP amplitude and latency	High; affected by attention, sleep, and drugs	Cortical registration, attention, development
MMN [8,20]	About 100–250 ms; auditory-cortical deviance response, with protocol-dependent frontal contribution	Passive oddball; deviant-minus-control difference	Variable; may persist when P3b is absent	Auditory regularity and sensory-memory testing
P3a/P3b [7]	About 250–600 ms; distributed frontoparietal networks	Novelty or active target/global rule	Very high	P3a: orienting; P3b: evaluation of task-relevant targets or global rules
40 Hz SSAEP [21,22,23,24]	Periodic; cortical/thalamocortical weighting	40 Hz modulation; complex-valued Fourier coefficients or coherent averaging	High; sensitive to drowsiness and anesthesia	State tracking and research audiology
FFR [25,26,27]	Sustained; cortical contribution increases at lower fundamental frequencies, and the cortical/subcortical balance also depends on montage	Periodic or quasi-periodic complex sounds; waveform, spectral, and phase analysis	Frequency-, montage-, and state-dependent	Representation of stimulus periodicity and spectral harmonics
High-rate SSAEP (about 80–100 Hz) [19]	Periodic; predominantly brainstem-weighted in adults	Faster-rate amplitude/frequency modulation; Fourier detection	Often more sleep-robust than 40 Hz; protocol-dependent	Objective threshold estimation

### 2.3. From Acoustic Event to Measured Waveform

An AEP is the product of the stimulus, auditory system, behavioral state, acquisition hardware, and analysis pipeline. Reports should specify whether stimulation is monotic (one ear only), diotic (the same signal to both ears), or dichotic (different signals to the two ears), as well as masking of the non-test ear when applicable. Click polarity, rise time, presentation level, interstimulus interval, and transducer delay also influence the input. Transducer delay is the interval between the electrical trigger recorded by the acquisition system and the arrival of sound at the ear canal. Electrode impedance, reference placement, amplifier bandwidth, digitization rate, and line-noise control influence the recording. Artifact rejection, filtering, baseline correction, averaging, peak measurement, and statistical thresholding influence the reported component. An apparent cross-state change can arise from the neural response, the background EEG signal, or a state-dependent interaction with the processing pipeline.

The distinction between near-field and far-field recording is also important. Intracranial contacts sample local or regional activity with high spatial specificity, whereas scalp electrodes record volume-conducted mixtures from multiple sources. Both electroencephalography and magnetoencephalography are orientation-dependent: magnetoencephalography is especially sensitive to tangential sources and relatively insensitive to radial sources, whereas electroencephalography detects both but weights them according to conductivity and reference geometry. Findings from EEG, magnetoencephalography, and intracranial recordings are therefore complementary rather than interchangeable.

Interpretive decisions associated with component detection should be separated from the cognitive claims made after detection. A statistically significant response at a prespecified latency or frequency establishes that the recording contains reproducible stimulus-related activity. Inferring hearing, attention, memory, or consciousness requires additional assumptions supported by the paradigm and comparison condition. Stronger cognitive claims require evidence that lower-level sensory responses were present, artifacts were excluded, and the relevant effect was reliably detectable in that individual.

## 3. Cognitive-State Modulation of AEPs

### 3.1. Wakefulness: Attention, Expectation, and Task Engagement

Wakefulness supplies the broadest range of top-down influences on auditory processing. In the classic selective-attention paradigm, identical tones elicited larger cortical responses when they were assigned to an attended rather than ignored stream [5]. Subsequent magnetoencephalography localized attentional modulation to auditory-cortical activity within approximately 20–50 ms in demanding dichotic-listening tasks [17]. These findings show that attentional selection can influence early cortical processing, although the latency and magnitude of the effect depend on stimulus rate, perceptual load, and the overlapping components being measured.

Attention does not uniformly increase the amplitude of every sound-evoked response. It changes the relative gain assigned to task-relevant features and streams. Human functional mapping has shown that sensory responses to acoustic features and attention-related modulations have partly distinct distributions across the auditory cortex, with stronger attention effects in lateral higher-order regions than in medial core regions [28]. At the scalp, this distributed reweighting can alter N1/P2 amplitude, sustained processing negativity, induced oscillations, and late target responses. A participant who stops following instructions may therefore produce a physiologically valid but cognitively different dataset.

A second mechanism is expectation based on acoustic regularity. Repetition establishes a short-term model of the acoustic environment, and a deviant can elicit a difference response even when the stream is unattended [8]. When the deviant is also task relevant, additional orienting and decision processes contribute to P3a and P3b. MMN and P300 should not be treated as successive magnitudes of a common process: they rely on different contrasts, have different task requirements, and can dissociate across states.

Awake recordings are sometimes treated as an uncomplicated baseline, yet they are often the most behaviorally variable condition. Vigilance, fatigue, motivation, hearing effort, medication, and mind wandering can change late AEPs over minutes. Strong studies verify audibility, document task performance, balance trial numbers, and model within-session drift. When the scientific question concerns state change, repeated measures within the same participant reduce interindividual variance but create additional concerns about learning, habituation, and order effects.

### 3.2. Natural Sleep: Preserved Monitoring Within a Reorganized Cortex

Sleep is not a uniform state of sensory shutdown. Early auditory conduction remains comparatively stable, whereas cortical responses are reorganized by sleep stage, ongoing slow waves, spindles, and the probability that a sound evokes a K-complex. The resulting waveform may not resemble a smaller waking ERP; it may contain sleep-specific components that overlap or replace waking components. Accurate staging from concurrent polysomnography is therefore essential. Pooling N1, N2, N3, and REM epochs into a single “sleep” condition can obscure the very physiology under study.

During NREM sleep, N1 amplitude can be markedly reduced or indistinguishable from baseline; latency estimates become unreliable when N1 is suppressed or overlapped by sleep-specific activity. P2, by contrast, can increase during both NREM and REM sleep [29]. During N2, sounds can evoke a large K-complex with a negative component near 500–600 ms followed by a positive deflection. Intracranial recordings identify the K-complex as a widespread cortical down-state rather than an enlarged N1 or P300 [30]. Its presence changes the interpretation of late averages, which should distinguish trials that do and do not evoke K-complexes whenever the question concerns residual stimulus discrimination.

Oddball studies demonstrate that differential processing can persist during behavioral unresponsiveness, but the form of the effect changes with stage. In eight volunteers, sleep stages II–IV produced K-complex-like responses to both frequent and deviant tones, with rare-tone responses four-to-fivefold larger, probably because of differential K-complex habituation. During REM sleep, a deviant-selective P3-like response with a waking-like topography reappeared [31]. In ten adults, the subject’s own name selectively enhanced the early K-complex during stage II and elicited a posterior P3-like response during REM sleep [32]. These findings support continued environmental monitoring, but they do not establish waking-like conscious access to every stimulus.

REM sleep has a desynchronized scalp EEG and reduced muscle tone, and auditory responses remain altered. Some mismatch-like or salience-related effects can be observed, whereas task-dependent P3b is unreliable because a sleeping participant is not maintaining the waking task instructions and response rule. Dream mentation and internal sensory activity may also compete with external input. Comparisons between REM and wakefulness should therefore control stimulus probability and arousal and should not assume that similar gross EEG appearance implies similar cognitive processing.

MLR effects are also component- and stage-dependent. In one adult natural-sleep study, Na and Pa latencies increased and amplitudes decreased as sleep deepened from stage II to stage IV, whereas REM values were similar to wakefulness [33].

SSAEPs reveal another level of state sensitivity. The seminal Galambos study characterized the 40 Hz response and noted prior reports of its disappearance under surgical anesthesia [21]. Direct studies subsequently showed reduced amplitude during drowsiness and sleep [24] and marked attenuation during selected general-anesthetic protocols [34]. Faster-rate responses used in hearing assessment may be more robust during natural sleep, but a protocol must not generalize the state behavior of one modulation frequency to all SSAEPs.

The overarching pattern is hierarchical, not absolute. Auditory nerve and brainstem conduction usually persist; thalamocortical and early cortical responses are attenuated or reshaped; automatic deviance and salience detection may survive under some conditions; and task-dependent target evaluation is the least reliable. Because spontaneous arousals can transiently restore waking-like responses, analyses should exclude or separately model epochs with microarousals and should report the time between stimulus and any sleep-stage transition.

**Figure 2:**
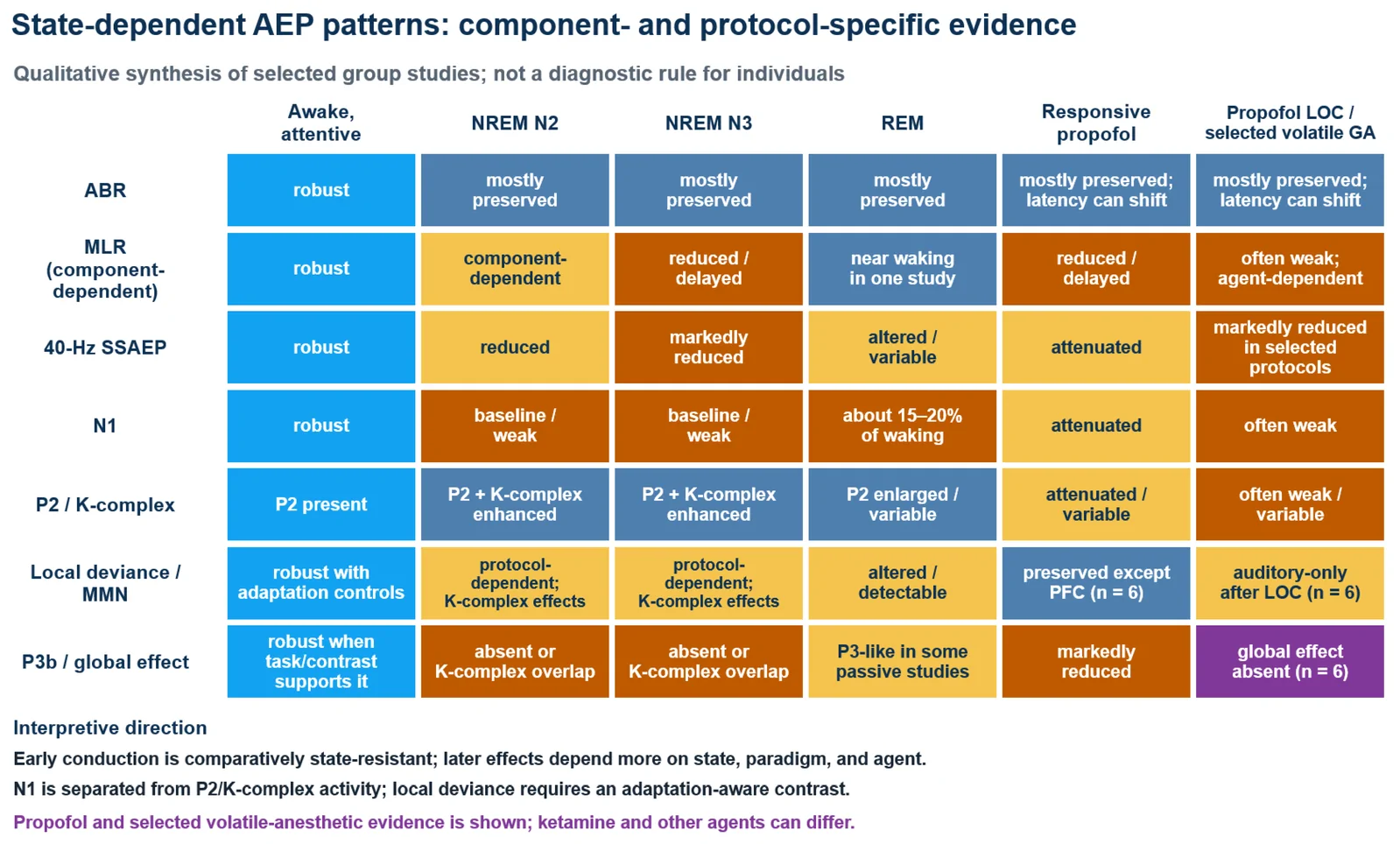
State-dependent AEP patterns. The cells summarize component- and protocol-specific group findings, including selected propofol and volatile-anesthetic studies; they are not individual diagnostic thresholds or universal drug effects. N1 is separated from P2/K-complex activity, and local deviance is separated from P3b/global-rule processing [24,29,30,31,32,33,34,35,36,37,38,39]. Ketamine can exert an opposing pharmacodynamic effect, including increased 40 Hz activity, and should not be generalized from the propofol and volatile-anesthetic pattern. Study-level evidence and protocol details are provided in Supplementary Table S1.

Compared with wakefulness, sleep generally preserves the ABR; attenuates or reshapes MLR, N1, and 40 Hz SSAEP measures; introduces K-complex-related late activity in NREM sleep; and reduces the reliability of task-dependent P3b. The exact profile remains stage- and protocol-dependent (Figure 2).

### 3.3. Sedation and General Anesthesia: Concentration- and Network-Dependent Effects

Sedation and anesthesia differ from natural sleep because drugs alter synaptic transmission and network dynamics in agent-specific ways. A useful design therefore reports the drug, target or measured concentration, co-administered analgesics, behavioral responsiveness, airway and physiologic variables, and the time course of induction and recovery. Terms such as “light,” “moderate,” and “deep” sedation are insufficient without an operational behavioral or pharmacologic definition.

Propofol studies illustrate the latency hierarchy. In nine surgical patients, propofol markedly affected middle-latency activity while producing smaller but measurable latency changes in ABR waves I, III, and V [35]. In 41 women, each studied at one of seven propofol concentration regimens, Nb latency and other MLR features varied with drug exposure and responsiveness; Nb latency discriminated eyelash response better than propofol concentration or dose rate [36]. Thus, the ABR is comparatively resistant and remains useful for pathway monitoring, but it is not invariant: temperature, anesthetic concentration, cochlear perfusion, and physiologic disturbance can still change its latency.

Late cognitive components are more vulnerable. During conscious propofol sedation, P3 amplitude fell substantially and reaction time increased, even though participants remained oriented and responsive [40]. Local–global auditory paradigms further show that propofol does not suppress the hierarchy uniformly. In six neurosurgical patients, local-deviance effects persisted outside the prefrontal cortex during responsive sedation, whereas global-deviance responses were markedly reduced and became absent after loss of responsiveness; at that point, local-deviance effects were restricted to the auditory cortex [37]. In eight patients with drug-resistant epilepsy studied during just-hypnotic propofol anesthesia, stimulus-evoked primary-auditory spiking and local field potentials persisted after loss of responsiveness, whereas higher-order spiking was largely attenuated. Induced gamma-band power did not always follow the local spiking response [38]. Together, these small intracranial studies support progressive restriction of auditory processing rather than complete cessation of sensory input.

The 40 Hz SSAEP and MLR have both been investigated as pharmacodynamic measures because they depend on synchronized thalamocortical and cortical activity. Under many propofol and volatile-anesthetic protocols, 40 Hz amplitude or power decreases as arousal is lost, and MLR latencies lengthen; however, neither response is a universal binary marker of consciousness. A response can be small because of montage, myogenic artifact control, hearing loss, poor stimulus delivery, or background EEG noise. A response can also persist despite unresponsiveness because some auditory circuits remain active. A controlled study showed that mastoid referencing can capture postauricular muscle activity and create misleading increases during drowsiness [24].

Surgical stimulation adds a further complication. In 30 cardiac-surgery patients receiving high-dose fentanyl plus propofol, isoflurane, or flunitrazepam, baseline MLRs differed by hypnotic regimen, but skin incision and sternotomy produced no significant additional MLR changes in any group [41]. The authors interpreted this lack of activation as consistent with blockade of surgical-stimulus effects by high-dose fentanyl. Thus, an AEP-based depth measure reflects hypnotic regimen within its analgesic and physiologic context; lack of activation does not independently prove adequate hypnosis. A monitor must also distinguish true cortical activation from electromyography, electrical interference, and changes in stimulus delivery.

Anesthetic-agent comparisons should not assume a common mechanism or response direction. Propofol and volatile anesthetics commonly suppress or delay cortical AEP measures, whereas ketamine can increase the 40 Hz response [39]. Nitrous oxide provides another dissociation: it hardly affected the middle-latency auditory evoked potential (MLAEP) even at concentrations that produced unresponsiveness, although xenon, isoflurane, and sevoflurane produced graded MLAEP changes during emergence [42]. Direct cross-agent comparisons using the same stimulus, behavioral endpoint, and analysis pipeline are therefore more informative than comparisons among laboratories using different methodologies.

Dexmedetomidine, an alpha-2 adrenergic agonist, produces a readily arousable, NREM-like sedative state that differs mechanistically from GABAergic anesthesia. In human intracranial recordings, dexmedetomidine and propofol both reduced local-deviance effects outside the auditory cortex around loss of responsiveness and suppressed global-deviance responses, while local effects persisted within the auditory cortex. These findings suggest partial convergence at the level of hierarchical auditory processing despite different receptor targets and emphasize that arousability does not make late contextual processing equivalent to wakefulness [43].

## 4. Human and Animal Studies

Animal models are indispensable because they allow recordings and causal manipulations at spatial scales that are rarely available in humans. The cat ABR generator experiments established how localized inactivation or lesions alter a scalp far-field response [11,12,13]. In behaving ferrets, task engagement rapidly changed spectrotemporal receptive fields in the primary auditory cortex [18]. In awake rats, epidural potentials to frequency and duration deviants included effects that could not be explained solely by stimulus-specific adaptation, while anesthesia changed the deviance response [44]. These studies provide mechanistic models for human state effects.

Translation is strongest when the measurement and computation are conserved. Auditory-nerve timing, brainstem conduction, tonotopy, onset responses, and some forms of deviance sensitivity have recognizable cross-species analogues and can be reasonably translated when the paradigm is matched. Absolute latencies do not translate directly because brain size, pathway length, cochlear mechanics, hearing range, and stimulus calibration differ. A “wave V” in one species cannot be equated with a human wave solely by ordinal position, and a rodent MMN-like difference should not be assumed to have the same relationship to conscious report as human P3b.

Anesthetic state is a major cross-species confound. Many animal auditory experiments are performed under agents that alter cortical gain, adaptation, oscillations, and long-range communication. A finding obtained under ketamine or isoflurane may describe the drug-shaped circuit as much as the awake circuit. Studies intended to explain human sleep or consciousness should include awake recordings when feasible, quantify state continuously, and test whether the relevant effect survives changes in anesthetic regimen.

Translation also requires species-specific acoustic calibration. Stimuli should be placed within the animal’s audibility range, and reports should document transducer coupling, middle-ear effects, body temperature, recording geometry, and the exact anesthetic protocol. Laminar organization and projection geometry can change which generator dominates a far-field peak. Shared ordinal labels therefore do not establish generator equivalence: a response called wave V in another species should be linked to the human wave V only through convergent lesion, latency, source, and pathway evidence [11,12,13,19,44].

Human intracranial studies bridge noninvasive human recordings and invasive animal experiments. They preserve human cortical organization and behavior while providing local field potentials, high-gamma activity, and sometimes single-unit recordings. Limitations include small clinically selected samples, nonuniform electrode coverage, anti-seizure medications, and the need to avoid epileptogenic tissue. Convergence among intracranial human recordings, noninvasive scalp AEPs, and causal animal experiments is therefore more persuasive than any one modality alone.

## 5. Pathway-Specific AEPs

### 5.1. Brainstem Responses and Frequency-Following Activity

The click-evoked ABR is the most clinically mature AEP because it is reproducible, rapid, and relatively resistant to changes in attention. Its principal measures are the presence and morphology of waves, absolute peak latency, interpeak latency, interaural differences, and amplitude ratios. Near threshold, wave V is usually the most robust component. At higher levels, waves I and V and the I–V interval help distinguish peripheral delay from central conduction abnormalities. Interpretation requires age- and protocol-appropriate norms because neonatal maturation, hearing loss, temperature, and stimulus rate influence timing.

“Brainstem response” should not be used as a synonym for “state independent.” The foundational human study found stable responses across repeated recordings and sleep [2], but anesthetic studies have documented smaller latency changes even when gross morphology remains usable [35]. The clinically relevant statement is that early waves are less state-sensitive than cortical AEPs under many common conditions, not that they are immune to state or physiology.

Complex sounds produce sustained responses that preserve periodic acoustic structure. In synthetic vowels, the human FFR represents the stimulus fundamental periodicity and distinct spectral peaks associated with vowel identity [25]. These measures can be collected without an active task and can characterize temporal fidelity under difficult recording conditions. However, the term “brainstem FFR” is not universally valid: cortical contributions become more prominent at lower fundamental frequencies and depend on montage [26,27]. Source claims should therefore be supported by stimulus frequency, latency, polarity behavior, and, ideally, source-sensitive recording.

In state research, brainstem responses provide an essential control. If an ABR or high-rate subcortical response is absent, a missing cortical response cannot be interpreted cleanly as loss of higher-order processing because the sound may not have reached the cortex effectively. Conversely, an intact ABR with absent P300 supports a dissociation between preserved ascending conduction and impaired context-dependent evaluation. This hierarchical control logic is one of the strongest reasons to combine early and late components in the same protocol.

### 5.2. Cortical Responses: N1/P2, MMN, and P300

N1/P2 reflects early cortical registration of acoustic onset and change. The N1 is a superposition of processes arising from supratemporal and association regions and is shaped by onset sharpness, presentation level, interstimulus interval, refractoriness, attention, and arousal [4]. A smaller N1 during sleep or sedation can reflect lower cortical gain, changed synchronization, overlap with slow activity, or altered adaptation. Peak amplitude alone cannot distinguish among these mechanisms.

MMN is a relational response. It is computed from the difference between responses to standards and deviants and therefore depends on how the standard regularity is established. Deviants can differ in frequency, duration, intensity, location, phoneme, or an abstract acoustic pattern. MMN is often described as preattentive because it can be observed when participants ignore the sounds [8], but attention, stimulus salience, and state can modulate it. A deviant-minus-standard difference also contains physical and adaptation differences unless the design includes appropriate control sequences.

The P300 family adds task and context. The original uncertainty experiments showed a late positive potential associated with resolution of stimulus uncertainty [7]. In contemporary oddball designs, P3a is often elicited by unexpected distractors and has a frontocentral distribution, while P3b is typically largest over parietal regions when a participant detects or categorizes a task-relevant target. The presence of a P3b-like global effect is stronger evidence of long-range, context-dependent processing than the presence of an early local deviance effect, but absence remains difficult to interpret in an individual because sensitivity is imperfect.

**Figure 3:**
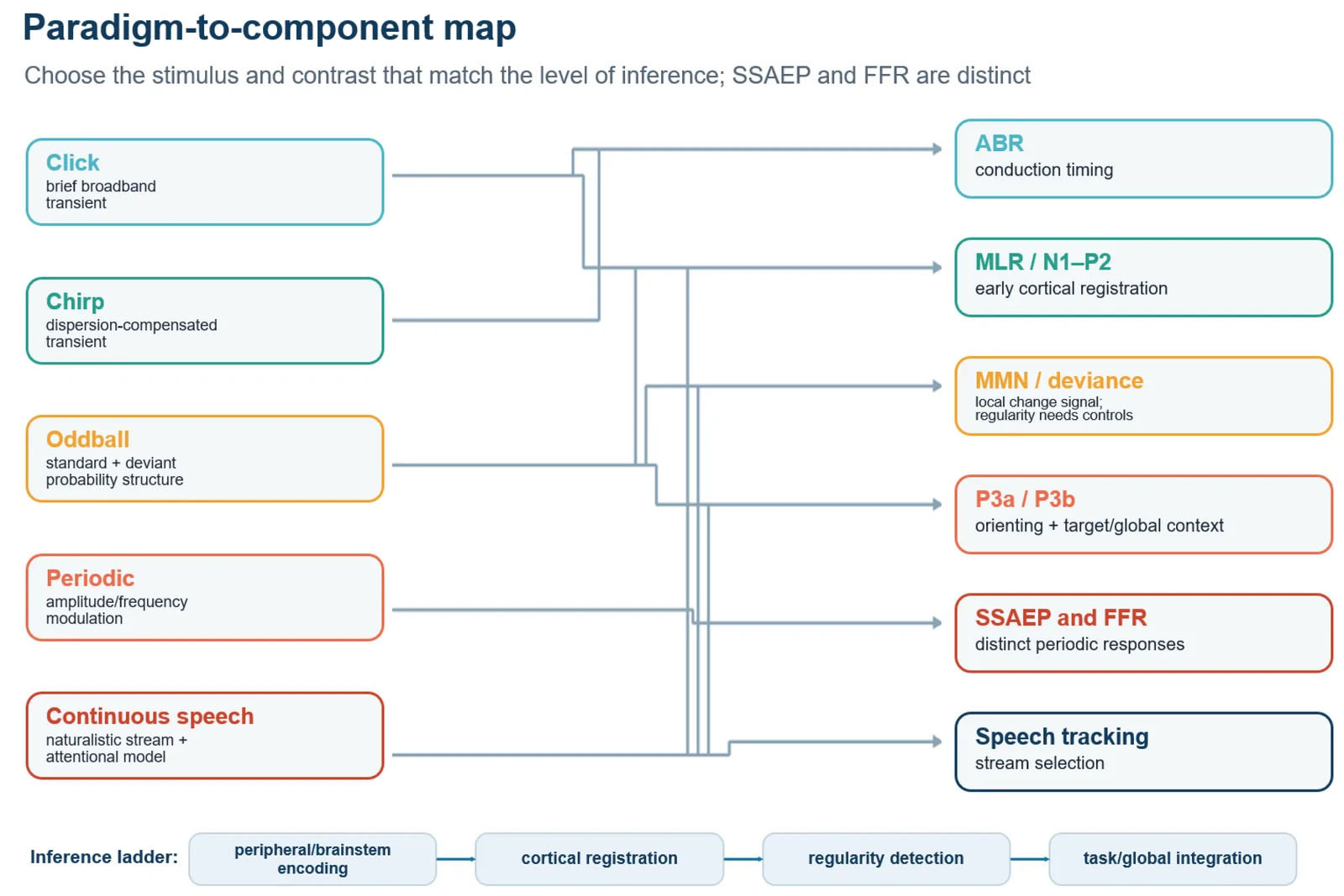
Paradigm-to-component map. Arrows show principal elicitation routes rather than exclusive relationships; inference becomes stronger only when earlier pathway levels and the relevant experimental contrast are verified.

These cortical components should be analyzed as a cascade rather than competing biomarkers. N1/P2 asks whether the cortex registered the event; MMN asks whether the event violated a regularity; P3a asks whether novelty recruited orienting; and P3b asks whether the event entered a task-defined context strongly enough to support updating or reporting. The stages overlap in time and anatomy, but the cascade makes explicit which inference is justified by each contrast. Figure 3 maps the principal stimulus paradigms to this component cascade and the inference supported at each level.

## 6. Stimulus Paradigms

### 6.1. Click-Evoked Responses

A click is a brief broadband transient that produces synchronized activation across a substantial portion of the auditory nerve. It is the canonical stimulus for ABR because its abrupt onset yields precisely timed early peaks [1,2]. Rarefaction, condensation, and alternating click polarity emphasize different cochlear and neural contributions; click polarity must therefore be reported. Insert earphones require correction for the acoustic delay between the transducer and ear canal, and contralateral masking may be needed when a high presentation level could stimulate the non-test ear.

The click’s strength is also its limitation. Cochlear traveling-wave delay means that high-frequency basal regions respond before lower-frequency apical regions, so the click does not activate the entire cochlea simultaneously. Its response is weighted toward the basal cochlea and does not provide frequency-specific threshold information without additional techniques such as tone bursts, masking, or derived-band analysis. Because the ABR is dominated by early conduction, a click is also poorly suited to testing auditory prediction or conscious evaluation unless it is embedded within a higher-order paradigm.

### 6.2. Chirp Stimuli and Cochlear-Dispersion Compensation

A transient sound does not reach all characteristic-frequency places on the basilar membrane at the same time. High-frequency energy reaches maximum excitation near the cochlear base earlier than low-frequency energy reaches more apical regions. The resulting dispersion spreads auditory-nerve firing over time and reduces the amount of activity that sums synchronously in the far-field waveform.

A rising-frequency chirp compensates for this delay by presenting low-frequency energy earlier and high-frequency energy later. Dau and colleagues designed an optimized chirp using a cochlear delay model so that excitation maxima across frequency regions would occur more nearly together [45]. The intended result is not that sound physically travels instantaneously through the cochlea, but that the timing of the stimulus counteracts the normal traveling-wave delay. More synchronized neural activation can produce a larger wave V than a conventional click, especially at low to moderate levels.

The optimal chirp delay pattern depends on stimulus presentation level because cochlear response latency changes with level. Elberling and Don therefore derived chirps from human derived-band ABR latencies and described a direct method for designing level-specific stimuli [46]. This development is clinically important: a chirp optimized at one presentation level can be suboptimal at another, and a larger amplitude does not automatically indicate better neural function. Reports should specify chirp family, presentation level, polarity, rate, calibration, and whether latency norms were established for that exact stimulus.

Chirps improve synchrony at the input to the ascending pathway; they do not make ABR immune to sleep, anesthesia, hearing loss, or neural dyssynchrony. Their benefit should be evaluated separately for response amplitude, detection time, threshold accuracy, and diagnostic specificity. When state is the independent variable, the same calibrated chirp and acquisition protocol must be used in every condition.

### 6.3. Oddball Paradigms: Probability, Regularity, and Task Relevance

The oddball paradigm is not merely a way to “produce MMN and P300.” It is a family of designs that manipulates probability, regularity, and task relevance. A frequent standard establishes a local model; an infrequent deviant violates one or more properties of that model. The participant may ignore the sequence, monitor it for targets, or learn a higher-order rule across groups of sounds. Each instruction changes the inference that can be drawn from the resulting waveform.

The classic P300 experiments manipulated stimulus uncertainty and associated its resolution with a late positive potential [7]. Later work distinguished an early negative deviance response from prolonged attention-related negativity that can overlap the N1 range [8]. In a passive oddball task, the deviant-minus-standard difference around 100–250 ms is commonly called MMN. In an active target task, a later P3b is expected when the participant detects, counts, or responds to the task-relevant target. Novel distractors may evoke P3a even when they are not the designated target.

Design choices strongly influence the measured components. Deviant probability determines how much evidence supports a standard regularity and how surprising the deviant is. The interstimulus interval influences refractoriness and sensory memory, and the magnitude and dimension of deviance affect discriminability. A duration deviant changes the physical time at which standard and deviant waveforms cease to be identical, whereas a frequency deviant differs from the standard beginning at acoustic onset. These properties determine the valid subtraction window and should be considered before a negative difference is interpreted as MMN.

Adaptation is the principal alternative explanation for a simple oddball difference. Neurons tuned to the frequent standard respond less after repetition, whereas neurons tuned to the rare deviant show less repetition-related adaptation. A many-standards or equiprobable control presents the deviant with the same probability but without a single repetitive standard. If the deviant still differs from its control, the evidence for regularity violation is stronger. Animal studies using such controls have demonstrated deviance-sensitive potentials beyond simple rarity under some awake conditions [44].

State changes alter both the component and the task. During wakefulness, an active oddball can separate early sensory registration, deviance detection, orienting, and target evaluation. During sleep or deep sedation, the participant cannot maintain the same overt task, so comparison with an awake active condition confounds state with instruction and behavior. Passive paradigms are better matched across states, but they usually provide weaker evidence for conscious access. A local-global design addresses this limitation by nesting short-range violations within longer-range sequence rules. In healthy awake participants, violations of a global rule evoke a late response associated with conscious processing [47]. In noncommunicating patients and during propofol anesthesia, local and global effects can dissociate [37,48].

An oddball result should therefore specify at least five elements: the physical standard and deviant; their probabilities; the sequence rule; the participant’s instruction and performance; and the contrast used to define each component. Reporting only “MMN was present” is insufficient. The same negative deflection could reflect adaptation, local deviance detection, attention-related processing, or overlap with a sleep-specific component.

### 6.4. Periodic Stimulation and Naturalistic Listening

SSAEP paradigms use tones, clicks, noise, or amplitude- and frequency-modulated carriers presented periodically. The neural response is evaluated at the stimulation frequency and, when present, its response harmonics. Multiple carriers can be modulated at different frequencies and presented simultaneously to estimate frequency-specific hearing sensitivity, but interactions, masking, and unequal signal-to-noise ratios require attention. Modulation rate shifts relative generator weighting: responses near 40 Hz contain strong auditory-cortical contributions, whereas responses near 80–100 Hz are relatively more brainstem weighted; neither range is source-pure [19].

Naturalistic paradigms trade tight event isolation for ecological validity. In competing-speech experiments, the neural response can be related to the temporal envelope of each speaker. Single-trial EEG has been used to decode which of two speakers a listener attends [49]. Such responses are not conventional peak-labeled AEPs, but they extend the same system-identification logic to continuous input. They are particularly promising for hearing devices and bedside communication assessment, provided that acoustic preprocessing, stimulus reconstruction, and temporal response functions are reported transparently.

## 7. Analysis Domains and Response Types

### 7.1. Acquisition, Preprocessing, and Reproducibility

AEP analysis begins before recording. The scientific question determines whether the protocol needs submillisecond ABR timing, wide-band cortical EEG, high trial counts, active behavioral responses, or uninterrupted sleep staging. The amplifier sampling rate and analog filters must preserve the component of interest. High-pass filtering can distort slow late potentials, and filter direction is critical: causal filters can shift or smear post-stimulus activity later in time, whereas acausal or zero-phase filtering uses future samples and can move energy before stimulus onset, creating artificial pre-stimulus deflections in difference waves and MMN windows. Low-pass filtering can remove sharp ABR features or induced high-frequency activity. Filter type, order, cutoff definition, causal direction, and application to continuous or epoched EEG data should be reported.

The reference electrode changes waveform amplitude and topography. Mastoid or earlobe references may be appropriate for some cortical ERPs but can capture postauricular muscle responses in 40 Hz work [24]. Common-average reference requires adequate scalp coverage. Linked references can introduce correlations and hide lateral asymmetry. Because no reference is electrically neutral, cross-state differences should be confirmed under a physiologically defensible montage rather than treated as invariant to rereferencing.

Artifact structure changes with state. Awake participants produce eye movements, blinks, speech, and muscle activity; sleeping participants produce slow eye movements, spindles, and stage transitions; surgical recordings include electrocautery, pumps, warming devices, and stimulus-delivery disturbances. Automated rejection thresholds applied identically across states may remove unequal proportions of data or preferentially reject physiologically large sleep responses. Reports should provide retained trial counts by condition and explain how ocular, muscle, line, stimulation, and movement artifacts were handled.

Consensus ERP guidance emphasizes prespecified components, explicit measurement windows, comparable trial counts, and complete reporting of acquisition and processing decisions [20]. These principles are particularly important in small clinical samples, where flexible peak selection can turn background fluctuations into apparent components. When possible, analysis code, de-identified summary data, and machine-readable event definitions should accompany the paper.

#### State-Aware AEP Minimum Reporting Checklist

1. Stimulus waveform, level, calibration, transducer, ear, polarity, rate, masking, and acoustic delay.
2. Electrode montage, reference, ground, impedance, amplifier, sampling rate, and recording bandwidth.
3. Analog and digital filters, including filter type, order, cutoff definition, causal direction, and whether filtering was applied to continuous or epoched data.
4. Ocular, muscle, line, stimulation, movement, and state-specific artifact handling.
5. Presented, rejected, and retained trial counts by condition and state, together with residual-noise or signal-quality criteria.
6. Operational state definition, behavioral responsiveness, sleep staging, arousal detection, and timing of state transitions.
7. Drug, dose or measured concentration, co-medications, infusion timing, physiology, and induction or recovery phase.
8. Audibility, hearing status, task instructions, behavioral accuracy, vigilance, and missing-response handling.
9. Prespecified component definition, measurement window, statistical test, multiplicity control, individual detection criterion, and treatment of indeterminate results.
10. Availability of analysis code, event definitions, de-identified summary data, and persistent source identifiers.

### 7.2. Transient, Phase-Locked Evoked Responses

Traditional ABR and ERP averaging assumes that the response recurs with consistent latency and phase relative to the onset of the acoustic stimulus while unrelated background EEG activity varies across trials. Averaging reinforces the repeated response and reduces uncorrelated noise. The resulting waveform can be measured by peak amplitude, mean amplitude within a time window, area, onset latency, fractional-area latency, or model-based component estimates. Each measure answers a different question and has different noise sensitivity.

Peak latency is intuitive for distinct ABR waves but unstable for broad or multi-peaked cortical components. Mean amplitude over a prespecified window is often more robust for MMN and P300, provided that the window is not chosen after inspecting condition differences. Difference waves can isolate condition-sensitive activity, but they combine noise and any physical differences between the contributing conditions. Topographic or source analyses require the same latency rule across electrodes; selecting a separate local peak at every channel can create a physiologically impossible map.

Single-subject detection demands a statistical criterion, not visual resemblance alone. Replication across independent trial subsets, residual-noise estimates, bootstrap confidence intervals, or frequency-domain tests can quantify reliability. A component visible in a group average may not be detectable in most individual participants, and a classifier trained on a group contrast may not be calibrated for a clinical decision about one patient. Sensitivity, specificity, test-retest reliability, and indeterminate results should be reported.

### 7.3. SSAEP Analysis

SSAEPs are phase-locked periodic responses and can be analyzed in the time or frequency domain. In the frequency domain, the primary question is whether energy and phase consistency at the stimulation frequency exceed the surrounding noise distribution. Measures include Fourier amplitude, signal-to-noise ratio, phase coherence, circular statistics, and multivariate combinations across channels. The analysis window should contain an integer number of modulation cycles or otherwise account for spectral leakage.

Time-domain averaging remains useful because it preserves phase and waveform shape. A controlled arousal study [24] found that 40 Hz response detection depended on analysis and montage; the spectral-averaging implementation used in that study was less efficient than time-domain averaging. Modern frequency-domain methods can preserve phase by averaging complex-valued Fourier coefficients. The general lesson is that “frequency-domain” does not guarantee optimal detection and that averaging power spectra is not equivalent to coherently averaging complex coefficients.

Throughout this review, SSAEP denotes the response class. If a cited paper uses ASSR in its title, that title is retained verbatim in the reference list, but the narrative term remains SSAEP. Reports should state carrier frequency, modulation frequency and depth, stimulus level, number and duration of epochs, phase treatment, rejection criteria, and statistical detection threshold. These details strongly influence both threshold inference and state sensitivity.

### 7.4. Phase-Locked Versus Non-Phase-Locked Activity

The phrase “non-phase-locked response” can be confusing because the response may still be stimulus-linked. The key distinction is between reproducibility of event timing and reproducibility of oscillatory phase. A phase-locked evoked response has a similar sequence of positive and negative voltage deflections on each trial. If its peaks align to stimulus onset, direct voltage averaging preserves it. ABR, MLR, N1/P2, MMN, P300, and SSAEPs are usually analyzed primarily in this way.

An induced oscillatory response can occur after the same event on every trial even when its instantaneous phase differs across trials. For example, a sound may increase power at 40 Hz between 200 and 400 ms. One trial may begin that interval with a positive gamma cycle and another with a negative cycle, so direct voltage averaging cancels the oscillation despite a power increase in both trials. Investigators recover such effects by calculating a time-frequency representation for each trial, using a wavelet transform, short-time Fourier transform, or related method, and then averaging power or another magnitude measure across trials.

Intertrial phase coherence and induced power answer different questions. Intertrial phase coherence measures whether oscillatory phase is consistent across repetitions. Power at a specified frequency, such as 40 Hz, measures the strength of the oscillation regardless of phase. A stimulus can increase both measures, increase phase coherence without much power change, or increase power without phase alignment. To isolate non-phase-locked activity, investigators sometimes subtract the phase-locked ERP from each trial before calculating power; this procedure has assumptions and can introduce bias.

Baseline normalization also matters. Decibel change, percent change, and subtraction can yield different results when baseline power differs across wakefulness, sleep, and anesthesia. A baseline contaminated by anticipatory activity or a preceding stimulus is not neutral. Time-frequency analyses also involve many time-frequency-electrode comparisons, so cluster-based or other multiplicity-aware inference should be prespecified. Tallon-Baudry and Bertrand’s distinction between evoked and induced gamma remains conceptually useful when implemented with these analytic cautions [50].

In plain language, phase-locked analysis asks, “Does the same waveform recur at the same point in every trial?” Induced-power analysis asks, “Does the event change the strength of an oscillation even if its peaks and troughs do not line up?” Both can be physiologically meaningful. They should not be placed in a table as mutually exclusive types of AEP without explaining that SSAEPs are phase-locked periodic responses, whereas induced activity is a broader stimulus-related oscillatory phenomenon.

### 7.5. Cross-State Comparisons

Cross-state inference is strongest when stimulus delivery, electrode geometry, preprocessing, and statistical detection remain constant while the state changes. Even then, background noise and artifact distributions may differ. Equalizing accepted trial counts, estimating residual noise separately, and reporting within-participant effect sizes reduce the risk that a smaller response in sleep or anesthesia merely reflects a noisier recording.

State should be measured rather than inferred from clock time or drug infusion alone. Awake tasks require performance and vigilance measures; sleep requires stage scoring and arousal detection; anesthesia requires concentration or dose history, behavioral responsiveness, and physiologic monitoring. For noncommunicating patients, diagnostic category, sedation exposure, etiology, time since injury, and repeated assessment are essential covariates.

## 8. Clinical and Translational Implications

### 8.1. Hearing Screening and Diagnostic Audiology

ABR supports objective assessment when a patient cannot provide a reliable behavioral threshold. In newborn programs, automated ABR and otoacoustic emissions identify infants who need diagnostic follow-up. Otoacoustic emissions are ear-canal sounds generated mainly by healthy cochlear outer hair cells and therefore assess cochlear, rather than neural, function. Universal newborn hearing screening is associated with earlier identification and may improve language and developmental outcomes, but a systematic review judged the certainty of evidence low or very low because the available studies were heterogeneous and vulnerable to bias [51]. The public-health benefit therefore depends on completion of diagnostic confirmation, amplification or other intervention, and language support—not on the screening waveform alone. Loss to follow-up and delayed diagnostic ABR remain practical barriers.

Frequency-specific tone-burst or chirp responses and SSAEPs can supplement click ABR when estimating an audiogram. Auditory neuropathy spectrum disorder illustrates why cochlear and neural measures must be combined: otoacoustic emissions or cochlear microphonics may show preserved outer-hair-cell function, while disrupted auditory-nerve synchrony produces an absent or distorted ABR. Sedation may facilitate recording but should be documented because it can influence cortical timing and, to a smaller degree, brainstem timing.

### 8.2. Intraoperative Monitoring

Intraoperative brainstem auditory evoked potential (BAEP) monitoring is used when surgery risks the cochlea, eighth nerve, or brainstem. The objective is continuous acquisition and rapid detection of a change that permits correction before permanent injury. Wave V latency, amplitude, and persistence, together with wave I and interpeak intervals, help distinguish a peripheral stimulus or cochlear problem from more central compromise. A prospective series of microvascular decompression procedures related progressive wave V delay and loss to postoperative hearing outcome and proposed graded warning values [52]. Warning thresholds from one procedure or laboratory should not be imported uncritically into another.

Mechanistic interpretation matters in real time. Loss of wave I suggests a problem before or at the distal nerve, poor stimulus delivery, or cochlear dysfunction; preserved wave I with delayed or lost wave V suggests more central conduction compromise. Hypothermia, hypotension, irrigation, drilling noise, anesthetic change, earphone displacement, and electrical interference can mimic neural deterioration. A monitoring response should therefore trigger a structured technical and physiologic check before a surgical conclusion.

### 8.3. Sedation and Anesthesia Monitoring

MLR, 40 Hz SSAEP, P300, and other auditory paradigms have been evaluated as measures of hypnotic state because they probe evoked cortical processing rather than spontaneous EEG alone. Commercial AEP-based depth monitors were developed and independently evaluated. In 22 volunteers exposed to desflurane and propofol, approximately 40 Hz power predicted responsiveness with a prediction probability of 0.96 under both agents, compared with 0.86 and 0.88 for Nb latency [53]. Propofol concentration-response studies also show systematic changes in midlatency features [36] and artificial neural networks have classified anesthetic states from MLR latency patterns more accurately than hemodynamic variables in a small surgical cohort [54]. These findings support continued development of AEP-informed monitoring, but not use of an auditory index as a standalone measure of consciousness.

Commercialization also exposed important limitations. In 13 patients, a composite A-Line index that combined MLAEP, spontaneous EEG, and burst-suppression information tracked calculated propofol effect-site concentration better than an earlier MLAEP-only index and was also compared with the bispectral index (BIS), although clinical performance remained to be established [55]. In ten awake, unmedicated volunteers, an earlier A-Line monitor produced index values from 15 to 99; 11.4% fell below the manufacturer’s range for surgical anesthesia because contaminated MLAEP signals were not reliably excluded [56]. No auditory measure should therefore be treated as a standalone consciousness meter. Hearing loss, stimulus disconnection, myogenic contamination, analgesia, and agent-specific pharmacology can all change the signal. A credible monitor should combine verified auditory input, early pathway integrity, a cortical evoked measure, spontaneous EEG, drug history, signal-validity checks, and clinical endpoints.

### 8.4. Disorders of Consciousness and the Intensive-Care Setting

Patients with coma, unresponsive wakefulness syndrome, or a minimally conscious state may have preserved sensory pathways but inconsistent motor output. A hierarchical auditory battery can test for evidence that sound activates the brainstem, reaches the cortex, violates a local regularity, and engages a global context. The local–global paradigm demonstrates the logic: healthy participants show early responses to local violations and a late effect to violations of a longer-range rule [47]. In patients with impaired consciousness, local effects may occur across diagnostic categories, whereas a late global effect is more specific but not sufficiently sensitive to rule out consciousness when absent [48].

Repeated measurements are crucial because arousal fluctuates; a single negative recording can result from noise, medication, hearing impairment, or a transient state. Prognostic studies should blind waveform interpretation to outcome and report indeterminate cases. The ethical implication is asymmetric: a reproducible late context-dependent response may provide positive evidence of preserved higher-order processing, but its absence should be interpreted cautiously.

A 2026 systematic review of 24 studies including 1562 adults found that absent brainstem or middle-latency responses often had high specificity but low sensitivity for poor outcome, whereas P300 findings were inconsistent. This pattern supports repeated, multimodal interpretation rather than using a single negative AEP to exclude residual function or favorable outcome [57].

ICU implementation also requires attention to competing sounds, ventilators, cranial injury, edema, hypothermia, and sedative exposure. Ear-specific delivery and an early response control help confirm effective auditory-pathway activation. Bedside paradigms should prioritize short acquisition, rapid analysis, and repeated single-patient reliability rather than maximizing the number of group-level components.

### 8.5. Developmental and Neuropsychiatric Applications

AEP latency and morphology change with maturation, so pediatric norms must be age specific. ABR maturation reflects myelination and conduction changes, while cortical P1, N1, P2, and mismatch responses follow distinct developmental trajectories. A developmental difference is not automatically a deficit; it may reflect age, sleep state, hearing experience, or stimulus familiarity.

Pediatric recordings also require state- and age-specific interpretation. ABR latencies shorten with maturation and myelination, cortical topographies and optimal SSAEP parameters change across infancy and childhood, and natural sleep can reduce movement artifact while simultaneously reshaping cortical responses and sleep-dependent plasticity. Studies should therefore report chronological and, for neonates, postconceptional age, hearing status, sleep stage or anesthetic, drug dose, temperature, and age-appropriate behavioral endpoints rather than applying adult norms.

Representative case-control studies have reported reduced 40 Hz SSAEP synchronization in schizophrenia, stimulus-dependent MMN and P3a differences in autistic children, and altered speech-evoked brainstem/FFR encoding in autism [58,59,60]. Effect direction and magnitude vary by stimulus and cohort. Clinical translation requires prospective individual-level validation, appropriate hearing controls, and demonstration that the measure adds value beyond behavioral and standard clinical assessment. Broad diagnostic labels should not be inferred from a single component. Table 2 summarizes the corresponding clinical uses, controls, and principal limitations.

### 8.6. Limitations of Clinical Translation

Clinical performance must be estimated against a prespecified reference standard and decision threshold. Sensitivity and specificity describe performance conditional on the true state, whereas positive and negative predictive values vary with prevalence, case mix, and the clinical setting. Studies should report confidence intervals, indeterminate tests, calibration, and incremental value over routine assessment rather than presenting group separation as an individual diagnostic threshold.

Absent responses are especially vulnerable to overinterpretation. Hearing loss, poor acoustic delivery, technical noise, medication, hypothermia, peripheral or brainstem dysfunction, and insufficient statistical sensitivity can all produce a negative result. A reproducible late response can provide positive evidence of preserved processing, but absence of that response does not prove absence of consciousness or recovery potential. Repeat testing, verified early-pathway responses, behavioral examination, spontaneous EEG, imaging, and other modalities should be integrated before individual decisions [57].

## 9. Limitations of the Review and Evidence Base

This narrative review was designed to integrate mechanistic, methodological, and clinical evidence rather than to estimate a pooled effect. The search and purposive selection may have missed relevant studies, and formal study-level risk of bias was not assessed. The underlying evidence is also heterogeneous: many anesthesia, sleep, intracranial, and disorders-of-consciousness studies are small; protocols, montages, state definitions, and endpoints differ; several clinically influential findings are old; and individual-level validation is limited. The synthesis should therefore be read as a framework for interpretation and study design, not as a quantitative practice guideline.

## 10. Future Directions

Naturalistic listening is likely to connect laboratory components with real-world hearing. Continuous-speech models can estimate how neural activity tracks attended and ignored speakers. A two-speaker study demonstrated that single-trial EEG could decode which speaker a listener attended [49]. Future work should combine this cortical tracking with verified peripheral or brainstem measures so that weak decoding can be attributed to the appropriate level of the auditory pathway.

Wearable systems can extend state research beyond short laboratory sessions. Around-the-ear and in-ear electrodes have captured N1- and P300-range effects during active hearing-device use [61], and c-shaped around-the-ear electrode grid (cEEGrid) configurations have also been evaluated for subcortical auditory potentials in normal-hearing and hearing-impaired listeners [62]. The remaining challenges include stable long-duration contact, motion and muscle artifact, accurate stimulus synchronization, user comfort, and validation against conventional montages.

Modern computational models should move from retrospective discrimination to transparent, calibrated decision support. Early work showed that nonlinear models could classify propofol anesthetic states from MLR features [54]. Models can now learn from time series or time-frequency representations, but high within-dataset accuracy does not establish clinical validity. Promising approaches include hierarchical calibration to an individual’s awake baseline, mixed-effects models that separate person- and state-level variation, and domain-adaptation methods that normalize differences among hospitals, amplifiers, montages, drugs, hearing profiles, and demographic groups. External validation remains essential. A clinical model should expose the signal features driving its decision and return uncertainty or “insufficient signal quality” rather than force every recording into a single state category.

Multimodal monitoring can test whether evoked and spontaneous measures provide complementary information. Propofol produces reproducible changes in spontaneous frontal EEG around loss and recovery of responsiveness [63]. Intracranial auditory studies show relative preservation of early primary-auditory responses and attenuation or spatial restriction of higher-order evoked and spiking responses; stimulus-induced power can also diverge from local spiking [37,38]. Combining these measurements may distinguish failure of sensory input, local cortical processing, and long-range integration. Such a system should be evaluated prospectively against behavioral responsiveness, explicit memory, and clinically relevant outcomes. In ambulatory and intensive-care settings, integrating continuous AEP/EEG with functional near-infrared spectroscopy or continuous pupillometry may provide convergent measures of cortical hemodynamics and arousal when any single modality is noisy or nonspecific.

Cross-species studies should use shared stimuli and analysis definitions. The same click, chirp, oddball, or amplitude-modulated waveform can be calibrated for species-specific hearing ranges while preserving the computational contrast. Awake animal recordings, causal perturbation, laminar physiology, and human intracranial data can then be connected to scalp biomarkers. Pre-registration of the predicted generator and direction of state effect would reduce post hoc mapping between unlike components.

Finally, the field needs a minimum reporting set for state-aware AEP research: calibrated stimulus waveform and transducer; ear and masking; electrode montage and impedance; state definition and monitoring; medications and concentrations; artifact and trial-retention statistics; filter and baseline parameters; component definition; individual-level detectability; and persistent identifiers for every cited source. Reference lists should include stable PubMed or DOI links so that readers can verify the underlying evidence efficiently.

## 11. Conclusions

AEPs should be interpreted as a hierarchy of related measurements rather than as a single test. Early ABR components provide a comparatively robust index of auditory-nerve and brainstem conduction. MLR and 40 Hz SSAEP measures probe thalamocortical and early cortical synchrony. N1/P2 reflects cortical registration, MMN tests sensitivity to auditory regularity, and P300 or global-rule effects provide stronger evidence of task- or context-dependent integration. Each level requires a stimulus and analysis matched to its generator.

Cognitive state determines which stages of the auditory hierarchy remain measurable and how response amplitude, latency, phase consistency, induced power, and spatial distribution change. Sleep reorganizes cortical responses and introduces stage-specific events. Sedation and anesthesia alter auditory processing according to agent, concentration, network level, and response measure; later and higher-order responses are generally more vulnerable than primary responses. Disorders of consciousness can preserve sensory activity despite absent behavioral output. None of these states is adequately summarized by “response present” or “response absent.”

The practical rule is simple: interpret a late component only after verifying the earlier pathway, and interpret any state difference only after verifying stimulus delivery, recording quality, analysis sensitivity, and the state itself. A multimodal, repeated, and component-specific approach offers the clearest route from auditory physiology to reliable biomarkers of hearing, arousal, and residual cognition.

## Figures and Tables

**Table 2:** Clinical translation matrix.

Application	Primary Response	Decision Supported	Essential Controls	Main Limitation
Newborn screening	Automated ABR with/without otoacoustic emissions	Referral for diagnostic hearing evaluation	Calibration, ear-specific testing, follow-up pathway	Screening does not establish etiology or guarantee intervention
Diagnostic audiology	Click, tone-burst, or chirp ABR; high-rate SSAEPs (approximately 80–100 Hz)	Threshold and pathway assessment	Age norms, masking, cochlear measures	Sedation, maturation, and neural dyssynchrony alter responses
Intraoperative monitoring	BAEP waves I and V; interpeak timing	Detect reversible cochlear/nerve/brainstem compromise	Stimulus integrity, temperature, blood pressure, artifact	Warning criteria vary by procedure and laboratory
Anesthesia monitoring	MLR, historically applied 40 Hz SSAEP, and late auditory ERP	Track drug-related changes in cortical and thalamocortical processing	Hearing, montage, drug/concentration, stimulation	No single component is a binary consciousness marker
Disorders of consciousness	ABR + N1/MMN + P3b/global effect	Identify preserved hierarchical processing	Repeated testing, medication, stimulus delivery and early pathway response, etiology	Positive late effects are informative; absence has limited sensitivity
Wearable monitoring	Ear-/around-ear N1/P300; ABR, FFR, and SSAEPs	Test feasibility of unobtrusive extended auditory monitoring	Synchronization, motion, contact stability	Clinical state decisions remain unvalidated; real-world signal quality varies

## Data Availability

No new data were created or analyzed in this study.

## Supplementary Materials

The following supporting information can be downloaded at: https://www.mdpi.com/article/10.3390/medsci14060618/s1, Table S1: Evidence Supporting Figure 2.

## Abbreviations

The following abbreviations are used in this manuscript:

AEP	Auditory evoked potential
ABR	Auditory brainstem response
ASSR	Auditory steady-state response
BAEP	Brainstem auditory evoked potential
BIS	Bispectral index
EEG	Electroencephalography
ERP	Event-related potential
FFR	Frequency-following response
ICU	Intensive care unit
MEG	Magnetoencephalography
MLAEP	Middle-latency auditory evoked potential
MLR	Middle-latency response
MMN	Mismatch negativity
NREM	Non-rapid eye movement
REM	Rapid eye movement
SSAEP	Auditory steady-state evoked potential
