# Supplementary material for: Auditory Evoked Potentials Across Cognitive States: Awake, Sleep, Sedation, and Anesthesia
Source: Med Sci (Basel). 2026 Sep 30;14(6):618. doi: 10.3390/medsci14060618 (PMC13706946; doi:10.3390/medsci14060618)
Supplement: Supplementary file 1 [file medsci-14-00618-s001.zip › medsci-4565181-supplementary.pdf]

Supplementary Table S1 Evidence Supporting Figure 2

This table summarizes the study-level evidence underlying the directional relationships shown in Figure 2.

Revised citation	Sample and species	State drug or protocol	AEP or component	Main direction relevant to Figure 2	Key qualification
[24] Picton 2003	Human; two experiments, n = 10 each	Awake, drowsy, and sleep	40-Hz auditory steady-state response	Response amplitude decreased with drowsiness and sleep.	Sensitive to electrode montage and artifact; the magnitude depends on recording conditions.
[29] Campbell 2011	Human; n = 10	Near-threshold tones during wake, NREM, and REM sleep	Cortical N1 and P2	N1 was absent in NREM and 15-20% of the waking response in REM; P2 was larger during sleep.	Small sample; near-threshold stimuli and sleep stage influence detectability.
[30] Cash 2009	Human; n = 8 with epilepsy; intracranial recordings	Stage 2 and slow-wave sleep	Intracranial K-complex-related responses	K-complexes coincided with widespread cortical down-states.	Epilepsy cohort; clinically determined electrode coverage limits spatial generalization.
[31] Bastuji 1995	Human; n = 8; drug-free	All-night auditory oddball across wake, stages I-IV, and REM	Auditory P300, K-complex, and P3-like response	P300 was attenuated in stage I; K-complexes dominated stages II-IV; a P3-like response appeared in REM.	Small overnight study; the observed component differed by sleep stage.
[32] Perrin 1999	Human; n = 10	Own name plus seven other names during stage II and REM	Name-evoked cortical AEPs	Selective responses to the participant's own name persisted in stage II and REM.	Personally salient stimuli; findings do not generalize to all sounds.
[33] Deiber 1989	Human adults; sample not stated in the abstract	Sleep stages II-IV and REM	Middle-latency Na and Pa	Na and Pa latencies increased and amplitudes decreased with sleep depth; REM approached waking values.	Sample size was not reported in the abstract; effects were sleep-stage specific.
[34] Plourde 1990	Human; n = 10; surgery	Thiopental, fentanyl, and isoflurane, with or without nitrous oxide	40-Hz auditory steady-state response	The response fell below background noise at surgical anesthesia and recovered during emergence.	Multiple agents and surgery prevent attribution to a single drug.
[35] Chassard 1989	Human; n = 9	Propofol 2 mg/kg induction; 7, 5, then 3 mg/kg/h	Middle-latency response and ABR	Propofol produced marked middle-latency changes but only modest ABR latency increases.	Small sample; the dose sequence and different response levels limit direct comparison.
[36] Tooley 1996	Human; n = 41 women	Blood propofol 1-10 micrograms/mL across seven regimens	Middle-latency response, including Nb latency	The response changed with concentration; Nb latency predicted loss of the eyelash reflex.	Women only; seven regimens and a surrogate endpoint.
[37] Nourski 2018	Human; n = 6 with epilepsy; intracranial recordings	Propofol from responsive sedation to loss of responsiveness	Local and global deviance responses	Global deviance was suppressed during sedation and absent at loss of responsiveness; local auditory deviance persisted.	Small epilepsy cohort; clinically determined electrode coverage.
[38] Krom 2020	Human; n = 8 with epilepsy; intracranial recordings	Propofol titrated to loss of responsiveness	Auditory cortical phase-amplitude coupling and spiking	Coupling and spiking persisted in lower auditory cortex while higher-level cortical responses attenuated.	Persistence of local responses does not demonstrate conscious access.
[39] Plourde 1997	Human; n = 12 plus 3 controls	Ketamine 1.5 mg/kg	40-Hz auditory steady-state response	The response increased, with no consistent relation to behavioral responsiveness.	Agent-specific paradoxical effect; small control group.

Note. All studies included human participants. Protocols, recording methods, and drug concentrations were heterogeneous; consult the full cited sources for complete methods and results.
